# The Effects of High Intensity Interval Training in Normobaric Hypoxia on Aerobic Capacity in Basketball Players

**DOI:** 10.2478/hukin-2013-0073

**Published:** 2013-12-31

**Authors:** Miłosz Czuba, Adam Zając, Adam Maszczyk, Robert Roczniok, Stanisław Poprzęcki, Wiesław Garbaciak, Tomasz Zając

**Affiliations:** 1 The Jerzy Kukuczka Academy of Physical Education in Katowice, Poland.

**Keywords:** intermittent hypoxic training, aerobic capacity, basketball

## Abstract

The aim of the present study was to evaluate the efficacy of 3-week high intensity interval training in normobaric hypoxia (IHT) on aerobic capacity in basketball players. Twelve male well trained basketball players, randomly divided into a hypoxia (H) group (n=6; age: 22±1.6 years; VO2max: 52.6±3.9 ml/kg/min; body height – BH: 188.8±6.1 cm; body mass – BM: 83.9±7.2 kg; % of body fat – FAT%: 11.2±3.1%), and a control (C) group (n=6; age: 22±2.4 years; VO2max: 53.0±5.2 ml/kg/min; BH: 194.3 ± 6.6 cm; BM: 99.9±11.1 kg; FAT% 11.0±2.8 %) took part in the study. The training program applied during the study was the same for both groups, but with different environmental conditions during the selected interval training sessions. For 3 weeks, all subjects performed three high intensity interval training sessions per week. During the interval training sessions, the H group trained in a normobaric hypoxic chamber at a simulated altitude of 2500 m, while the group C performed interval training sessions under normoxia conditions also inside the chamber. Each interval running training sessions consisted of four to five 4 min bouts at 90% of VO2max velocity determined in hypoxia (vVO2max-hyp) for the H group and 90% of velocity at VO2max determined in normoxia for the group C. The statistical post-hoc analysis showed that the training in hypoxia caused a significant (p<0.001) increase (10%) in total distance during the ramp test protocol (the speed was increased linearly by 1 km/h per 1min until volitional exhaustion), as well as increased (p<0.01) absolute (4.5%) and relative (6.2%) maximal workload (WRmax). Also, the absolute and relative values of VO2max in this group increased significantly (p<0.001) by 6.5% and 7.8%. Significant, yet minor changes were also observed in the group C, where training in normoxia caused an increase (p<0.05) in relative values of WRmax by 2.8%, as well as an increase (p<0.05) in the absolute (1.3%) and relative (2.1%) values of VO2max. This data suggest that an intermittent hypoxic training protocol with high intensity intervals (4 to 5 × 4 min bouts at 90% of vVO2max-hyp) is an effective training means for improving aerobic capacity at sea level in basketball players.

## Introduction

Basketball is an intermittent sport discipline, which is predominantly anaerobic in nature. During a basketball game at the international level of competition, a player covers from 3500 to 6100m, depending on the position and the tactics of the game. High intensity activities usually last from 1 to 4s and occur on the average every 20–25s. These periods are interspersed with activities of low to moderate intensity like jogging, walking, standing, inbound passing or free throw shooting. The intensity of the game changes very rapidly and some authors have shown that players perform over 1000 changes of direction and pace during a game (McInnes et al., 1995). The high intensity activities are powered by the PCr system, which is resynthesized via aerobic metabolism during the short frequent breaks (time outs, out of bounds, free throws etc.). Aerobic capacity in elite basketball players varies significantly depending on the court position. Guards and small forwards have VO_2max_ values in the range of 54–62 ml/min/kg, while centers and power forwards’ oxygen uptake is within the range of 48–54 ml/min/kg (Abdelkrim et al., 2007). Castagna et al. (2008) showed a significant relationship between aerobic capacity and the rate of recovery, during short rest periods in basketball players. A high level of aerobic fitness enhances the recovery process in basketball players during the short breaks and periods of reduced activity, thus allowing the athlete to perform numerous explosive movements throughout the game without compromising the quality of performance.

The concept of altitude or hypoxic training is a common practice for improving aerobic capacity and endurance performance. After 40 years of experimenting with this training method, several strategies of training have been proposed, like “live high - train high” (LH-TH), “live high – train low” (LH-TL) or “intermittent hypoxic training” (IHT). The first two strategies (LH-TH and LH-TL) are based on adaptive changes of humans to chronic hypoxia. Chronic exposure to moderate altitudes (2,000–3,000 m) improves oxygen transport capacity by enhancing erythropoietin secretion and the consequential increase in total hemoglobin mass (Bunn and Poyton, 1996). This results in augmented maximal oxygen uptake (VO_2max_) and enhanced exercise performance. These adaptations can be seen after 2 to 3 weeks of exposure to moderate altitudes.

During the last decade, significant attention in sport sciences has been given to IHT, which theoretically, could be an effective training method for improving aerobic capacity and endurance performance. In this method, athletes live under normoxic conditions and train in a natural hypobaric or simulated normobaric hypoxic environment. Theoretically, the stress of hypoxic exposure, in addition to the training stress, will foster the adaptations experienced with endurance exercise and will lead to greater improvements in performance. The improvement in low altitude (“sea-level”) performance and an increase in VO_2max_ after IHT cannot be explained by changes in blood variables alone, but is also associated with nonhematological adaptive mechanisms, which are also observed after chronic hypoxia (Czuba et al., 2011). The results of our previous study (Czuba et al., 2011) and other well-controlled studies (Dufour et al., 2006; Zoll et al., 2006) indicate that the improvements in aerobic capacity and endurance performance are caused by muscular and systemic adaptations, which are either absent or less developed after training under normoxia. IHT may cause more pronounced adaptive changes in muscle tissues in comparison to traditional endurance training under normoxic conditions. These changes include increased skeletal muscle mitochondrial density, elevated capillary-to-fiber ratio, and increased fiber cross-sectional area (Desplanches and Hoppeler, 1993; Vogt et al., 2001).

Current research results on the effectiveness of IHT for improving VO_2max_ and endurance performance at sea-level are ambiguous. Only a few studies (Terrados et al., 1990; Melissa et al., 1997; Green et al., 1999; Zoll et al., 2006; Robertson et al., 2010; Czuba et al., 2011) reported enhanced endurance performance at sea-level following IHT, while a number of research projects have failed to demonstrate improvement in sea-level performance after IHT (Truijens et al., 2003; Ventura, 2003; Morton and Cable, 2005; Roels et al., 2007).

These conflicting reports on the efficacy of IHT may be due to methodological differences, including the type, volume and intensity of exercise in the hypoxic environment, as well as the intensity of the hypoxic stimulus and the sports level of research subjects.

According to the review of Bonetti and Hopkins (2009), exercise intensity is reported to be a major factor determining the magnitude of changes when training is performed in hypoxia. They conclude that moderate-intensity training (close to AT) more efficiently increases performance (Dufour et al., 2006; Zoll et al., 2006, Czuba et al., 2011) than higher exercise intensities (close to or equal to maximal aerobic power (Roels et al., 2005, 2007). There are also reports demonstrating no beneficial effects of IHT programs on aerobic capacity or endurance performance, when the intensity during IHT sessions was set below 80% of VO_2max_ at sea level (Truijens et al., 2003; Ventura et al., 2003). Ventura et al. (2003) reported only a non-significant tendency for increased values of maximal power output (∼4%) in the IHT in relation to the placebo group. However, in one of the most recent studies conducted by Hamlin et al. (2010), the authors found a small (2–3%), difficult to explain improvement in a 20 km time trial average power output in the IHT compared with the placebo group.

We hypothesized that not only moderate or high intensity of the exercise improves VO_2max_ during IHT protocol or normoxia, but also the total volume of training at this intensity during a single work-out and the whole cycle (3–4 weeks). The research of Helgerud et al. (2007) seems to back up our hypothesis related to the intensity and volume of work performed under normoxia. They analyzed the effect of four different training methods (long slow distance running at 60% of VO_2max_, lactate threshold running at 80% of VO_2max_, 15/15 interval training at 87.5% of VO_2max_, and 4×4 min interval training at 87.5% of VO_2max_) in normoxia on VO_2max_ and stroke volume. All four training protocols resulted in a similar total oxygen uptake and were performed 3 days per week for 8 weeks. They found the highest improvements in VO_2max_ and stroke volume after the 4 × 4 min of interval running (4 min of running at 87.5% of VO_2max_ followed by 3 min of active rest, jogging at 60% of VO_2max_).

Very little research with IHT has been performed on team sports players where aerobic power and capacity play a significant role in game performance. Based on the above observations, we attempted to test the hypotheses that an innovative IHT program, including three weekly high intensity hypoxic exercise bouts of 4 minute duration, improves aerobic capacity in basketball players at sea-level.

## Material and Methods

### Participants

Twelve male basketball players, with at least 5 years of systematic training experience took part in this study. All of the tested athletes possessed current medical examinations, stating no contradictions to perform exhaustive exercise in a hypoxic environment. All subjects were randomly divided into a hypoxia (H) group (n=6; age: 22±1.6 years; VO_2max_: 52.6±3.9 ml/kg/min; body height – BH: 188.8±6.1 cm; body mass – BM: 83.9±7.2 kg; fat content - FAT%: 11.2±3.1%), which trained in a normobaric hypoxia environment, and a control (C) group, which exercised under normoxic conditions (n=6; age: 22±2.4 years; VO_2max_: 53.0±5.2 ml/kg/min; BH: 194.3±6.6 cm; BM: 99.9±11.1 kg; FAT%: 11.0±2.8 %).

The experiment was conducted according to the Declaration of Helsinki, with a clear statement that all of the participants were informed of the objectives of the research, possible risks and could withdraw at any time of the study. The research project was approved by the Ethics Committee for Scientific Research at the Academy of Physical Education in Katowice, Poland.

### Experimental Design

The research was conducted during the specific phase of the preparatory period (September, 2012) after 4 weeks of general conditioning. The experiment had two series of testing, separated by four microcycles of training (24 days). The first series of testing was conducted at the beginning of the experiment to determine initial values of analyzed variables. Two days before the first series of testing, all participants were familiarized with the testing procedure. Three microcycles (3 weeks) with progressive training loads were then applied, followed by a fourth short recovery microcycle (3 days). After the recovery microcycle, the last series of testing was peeformed. The testing procedure in the second series was the same as in the first one.

### Testing Protocol

This research project had two series (S1, S2) of testing in the laboratory, between 4 and 2 days pre- (S1) and 3 days post training (S2). Both series of testing (S1, S2) included one day of investigations in normoxia. Additionally, 2 days before the training program was initiated (during S1), all athletes in the H group performed the same test in hypoxia conditions to establish individual training loads for IHT sessions.

On the first day of each series of testing (S1,S2), before breakfast after an overnight fast, resting blood samples were drawn from the antecubical vein to determine hematological variables (hemoglobin concentration (HGB), haematocrit value (HCT), number of erythrocytes (RBC) (Advida 2120, Siemens, Germany). Body mass and body composition were then evaluated by electrical impedance (Inbody 720, Biospace Co., Japan). Two hours after a light breakfast (Carbohydrates – 50%, Protein −20%, Fats – 30%), a ramp treadmill test was administered to determine aerobic capacity.

The test was performed on a Pulsar treadmill (HP-Cosmos, Germany), beginning at 6 km/h and 0 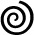 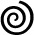 inclination. Treadmill speed was increased linearly by 1 km/h per 1min (0.016 km/h per 1s) until volitional exhaustion. During the test, heart rate, minute ventilation (VE), oxygen uptake (VO_2_) and expired carbon dioxide (CO_2_) were continuously measured using a MetaMax 3B telemetry spiroergometer (Cortex, Germany) in the breath-by-breath mode. VO_2max_ was determined based on decreased or a plateau in VO_2_ at rising speed ( 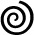
VO_2_ ≤ 150 mL/min at VO_2peak_). Fingertip capillary blood samples for the assessment of lactate (LA) concentration (Biosen C-line Clinic, EKF-diagnostic GmbH, Germany) were drawn at rest and at the end of each test, as well as during the 3^rd^, 6^th^, 9^th^, and 12^th^ min of recovery. Also, capillary rest and post-exercise blood samples were used to determine acid-base equilibrium and oxygen saturation of hemoglobin (RapidLab 248, Bayer Diagnostics, Germany).

After 48h of rest, athletes in the H group performed the same ramp test protocol in normobaric hypoxia conditions (LOS-HYP_1/3NU; LOWOXYGEN® SYSTEMS, Germany) equivalent to 2500 m altitude (F_I_O_2_=15.2%) to establish individual training loads for IHT sessions.

The atmospheric conditions in regard to temperature (18,9°C – S1; 19,2°C - S2) and humidity (51% - S1; 52% - S2) were held constant in both series of testing to increase the reliability of measurements.

### Training program

The training program applied during the experiment was the same for both groups, but with different environmental conditions during the selected morning interval training sessions. All of the research participants were members of a single basketball team, which practiced 6 days per week in the afternoon, with each session lasting from 90 to 120min. These specific training sessions included technical and tactical drills, with days of low and high training loads, which were alternative to the IHT. For three microcycles (three weeks), all subjects preformed three high intensity interval training sessions per week. During the interval training sessions, the H group trained in a normobaric hypoxic chamber at a simulated altitude of 2500 m (FIO2=15.2%). The C group preformed interval training sessions in the same normobaric hypoxic chamber but under normoxia conditions. During all interval training sessions, the atmospheric conditions in regard to temperature (19°C), humidity (50%), and concentration of carbon dioxide (700–800 ppm) were controlled and held constant to increase the reliability of the investigations. During each interval training session all participants in the H and C group drank *ad libitum* the same electrolyte drinks.

The intensity for the interval training sessions was calculated individually for each athlete in both groups. In the H group, the intensity was calculated upon the % of velocity at VO_2max_ determined in hypoxia (vVO_2max_-hyp), however, in the group C it was based at % of velocity at VO_2max_ determined in normoxia (vVO_2max_).

Each interval training session consisted of four to five 4 min bouts at 90% of vVO_2max_-hyp / vVO_2max_ (H group / C group) separated by 4 min of active recovery at 60% of vVO_2max_-hyp / vVO_2max_ (H group / C group). Before performing the four bouts, athletes in both groups performed a 15 min warm-up. The warm-up intensity was set at 60% of vVO_2max_-hyp/vVO_2max_ for its first 10 minutes and 70% of vVO_2max_-hyp/vVO_2max_ for its last 5 minutes. After the interval session, athletes in both groups performed a 10 min cool-down, at an intensity equivalent to 60% of vVO_2max_-hyp/vVO_2max_. The volume of training during the interval sessions in both groups was increased from 4 to 5 bouts after the second microcyle. Besides registering the intensity and volume of the training process, at the beginning of each microcyle, and after one day of rest, blood samples were drawn from the antecubical vein to determine changes in hematological variables (HGB, HCT, RBC). Also pre- and post interval training capillary blood samples were drawn to determine LA, acid-base balance and saturation of hemoglobin to determine possible changes in these variables.

### Statistical Analysis

The obtained data were analyzed statistically with the use of Statistica 10.0 (StatSoft). Basic descriptive statistics were calculated, and all variables were examined for normal distribution. To determine the influence of intermittent hypoxia training (IHT) on aerobic capacity in normoxia, the two-way ANOVA (group & training) with repeated measures was applied. When significant differences in F ratio were found, the *post hoc* Tukey’s test was used. The level of statistical significance was set at p<0.05.

## Results

The average values of body mass and body composition, as well as chosen hematological variables are presented in Tables 1 and 2. The ramp test results and values of chosen physiological and biochemical variables obtained, as well as the significance of differences between both series of testing during the experiment are presented in Table 3.

A two-way analysis of variance showed a statistically significant effect of the two main factors (group & training) on registered variables during the ramp treadmill test, such as: total distance covered during the ramp test (F=14.268, p=0.003), maximal workload (WR_max_; F=6.429, p=0.029), maximal oxygen uptake (VO_2max_, F=80.192, p=0.001), maximal heart rate (HR_max_; F=5.914, p=0.048), maximal oxygen pulse (O_2_/HR_max_; F=65.533, p=0.001), delta (Δ) values of lactate concentration during the test (ΔLA; F=5.441, p=0.049) and delta (Δ) values of lactate concentration during the 12min of recovery (ΔLA12′rec; F=9.442, p=0.012). The training program used in this research did not significantly affect the analyzed hematological variables (RBC, HGB, HCT, MCV) (Table 2), as well as body mass and body composition (Table 1).

### Post hoc analysis

**Figure 1:**
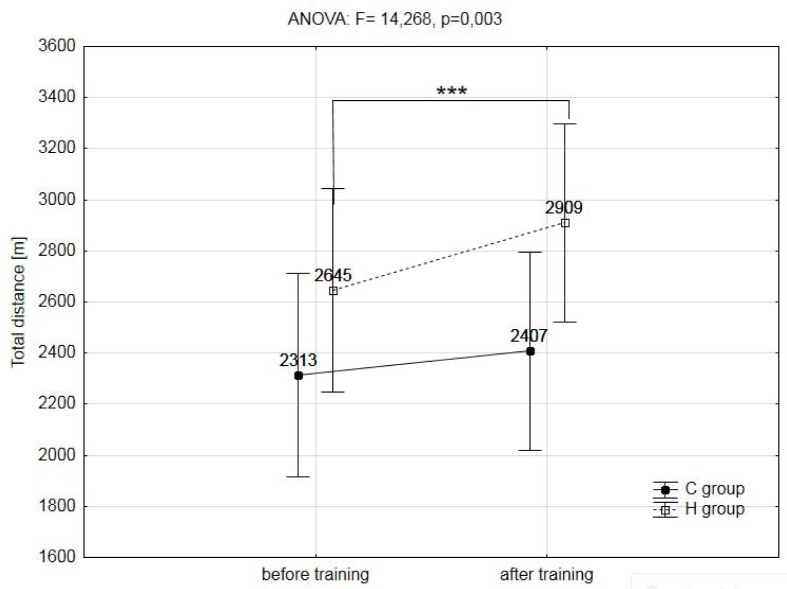
The total distance during the ramp test protocol in hypoxic (H) and control (C) groups before and after training; *** - p<0.001

**Figure 2:**
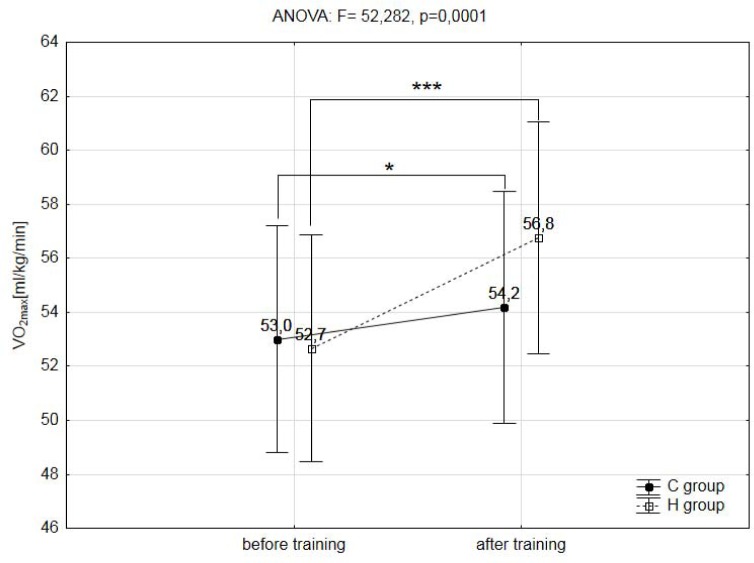
The relatives values of the maximal oxygen uptake (VO_2max_) observed during the ramp test in hypoxic (H) and control (C) groups before and after training; ** - p<0.01

The post-hoc analysis showed that the IHT caused a significant (p<0.001) increase in total distance covered during the ramp test protocol by 10% in the group H (Figure 1), as well as a 4.5% increase (p<0.01) in the absolute maximal workload (WR_max_) and 6.2% (p<0.01) in relative values of this variable. Also, the absolute and relative values of maximal oxygen uptake (VO_2max_) in this group increased significantly (p<0.001) by 6,5% and 7,8% (Figure 2).

Similar but minor changes were also observed in the group C. The post-hoc analysis showed that the interval training in normoxia caused a significant (p<0.05) increase in relative values of WR_max_ by 2.8%, as well as an increase (p<0.05) in absolute and relative values of VO_2max_ by 1.3% and 2.1% respectively (Figure 2).

**Figure 3:**
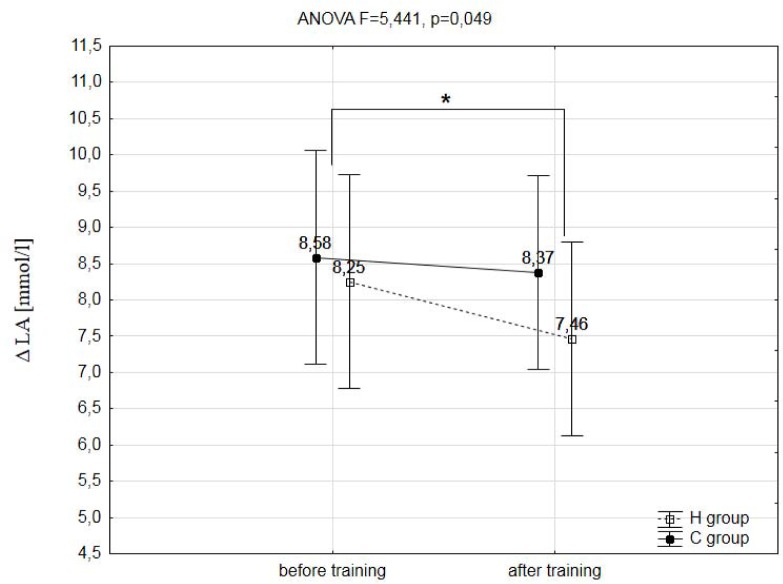
The increase in the blood lactate concentration after ramp test (ΔLA) in hypoxic (H) and control (C) groups before and after training; * - p<0.05

**Figure 4:**
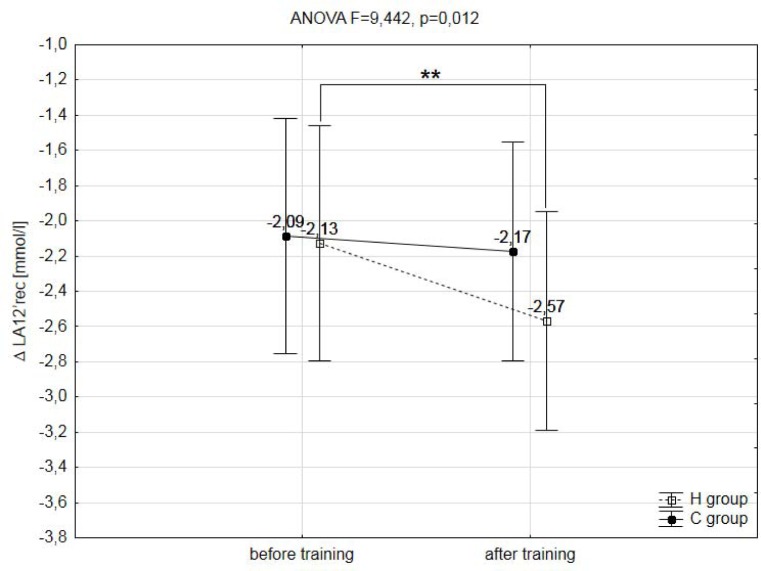
The decrease in the blood lactate concentration observed 12 minutes after the end of the ramp test (ΔLA12′rec) in hypoxic (H) and control (C) groups before and after training; ** - p<0.01

However, a small but significant (p<0.05) decrease of 1.6% in maximal heart rate (HR_max_) was observed only after the IHT. A significant (p<0.001) increase in VO_2max_ and a (p<0.05) decrease in HR_max_ caused a significant (p<0.001) increase in maximal oxygen pulse (O_2_/HR_max_) in the group H after three weeks of IHT. Also, the delta values in lactate concentration after the ramp test (ΔLA) were in the group H significantly (p<0.05) lower in comparison to baseline levels by 9.5% (Figure 3). However, the rate of LA utilization after the 12 minutes of recovery (ΔLA12′rec) were significantly (p<0.01) higher after IHT (Figure 4).

## Discussion

The most important finding of this study is that a 3 week IHT program, including three weekly high intensity hypoxic exercise bouts of 4 minutes duration, caused a significantly higher improvement in VO_2max_ as well as, in relative maximal workload (WR_max_) than the same training procedure performed under normoxia conditions (VO_2max_: 7.8% - H group vs. 2.1% - C group; WR_max_: 6.2% - H group vs. 2.8% - C group). Also, the results of this study show a significantly increased (10%) total distance covered during the ramp test protocol after the IHT procedure.

Theoretically, intermittent hypoxic training (IHT) may increase aerobic capacity which is most often evaluated by maximal oxygen uptake (VO_2max_), as well as improve endurance performance at sea-level by several adaptive changes. However, current research findings on IHT as an effective method for enhancing aerobic capacity and sport performance at sea-level are inconclusive.

Accordingly, improvement of sport performance and VO_2max_ in swimmers has not been observed after an IHT program, including very short intervals (60s and shorter) with high intensity (Truijens et al., 2003). Similarly, Morton and Cable (2005) observed no significant differences in values of VO_2max_ between IHT and control groups, after short interval (60s) training sessions during IHT program. However, the longer high intensity intervals (2min) in conjunction with low intensity continuous exercise (60% VO_2max_) performed during IHT sessions improved only maximal power output at sea level in male endurance athletes, without increases in VO_2max_ (Roels et al., 2007). In another study, Roels et al. (2005) observed a significant improvement in VO_2max_ after intermittent hypoxic interval training (IHIT), where athletes performed the warm-up, the cool-down, and the recovery from each interval in hypoxia, whereas the high-intensity exercise bouts of 2 to 12- minute duration were in normoxic conditions. Despite the significantly improved VO_2max_, the results did not show an improvement in mean power generated during 10 minute individual time trial after the IHIT protocol was applied; the hematologic indicators did not change either. However, they did not observe an improvement in VO_2max_ after the same training program performed in hypoxia conditions (typical IHT) and normoxia.

In addition, the results of our last study (Czuba et al., 2011) allow us to conclude that IHT for three weeks (3 IHT sessions per week) with prolonged exercise (30–40min) at lactate threshold is an effective training means for improving VO_2max_ and endurance performance at sea-level. This results are in accordance to those obtained by Dufour et al. (2006) and Zoll et al. (2006), where subjects trained with very similar intensity (at the second ventilatory threshold) during IHT sessions for six weeks, but twice a week.

A comparison of the results of these experiments (Dufour et al., 2006; Zoll et al., 2006; Czuba et al., 2011) with those obtained by the authors suggests that exercising during IHT at the anaerobic threshold intensity selected individually to match the designated altitude (hypoxia) effectively improves aerobic capacity and exercise performance.

Similarly, Robertson et al. (2010) observed a significant improvement in values of VO_2max_ after 3 weeks of IHT protocol (4 training sessions per week at 2,200 m). The IHT sessions incorporated one long, one moderate duration, and two interval sessions with high intensity per week. The specific content of these sessions was based on individual training programmes with athletes instructed to complete between 4 and 5 h of hypoxic training per week, depending on their normal training load. Unfortunately, the authors did not report more details about the duration of IHT sessions.

There is also strong evidence that demonstrated no beneficial effects of IHT programs on aerobic capacity, when the intensity during IHT sessions was set below 80% of VO_2max_ at sea level (Vallier et al., 1996; Truijens et al., 2003; Ventura et al., 2005). The absence of positive adaptive changes in these athletes is very probable due to insufficient exercise intensity during the IHT protocol. The recent study on IHT that was carried out with triathletes placed in a hypobaric chamber (Hendriksen and Meeuwsen, 2003) also failed to demonstrate improvements in VO_2max_. Exercise intensity in that study was selected individually to correspond to 60–70% of Heart Rate Reserve (HRR), so the subjects exercised in the aerobic exercise zone during a 10-day training period. During the annual training cycles, such training units are used to maintain the athlete’s fitness level and not to improve it.

The results of this study build on and enhance the earlier research into the IHT method. In the analysed basketball players, significantly higher VO_2max_ (by 8%) and longer distance covered in the maximal ramp test were recorded after 3 weeks of high intensity interval training in normobaric hypoxia. In the group H where the IHT protocol was applied, the increase in VO_2max_ was greater by 5% than in the group C that trained in normoxia.

Considering the findings of this study and earlier investigations (Vallier et al., 1996; Truijens et al., 2003; Morton and Cable, 2005; Roels et al., 2005, 2007; Dufour et al., 2006; Zoll et al., 2006; Hamlin et al., 2010; Czuba et al., 2011) we are in agreement with Bonetti and Hopkins (2009) report, that intensity of the training is a major factor determining the magnitude of changes when training is performed in hypoxia. We also conclude that not only the intensity of exercise influenced the improvements in VO_2max_ during the IHT protocol, but also the total volume of training at this intensity during a single work-out.

In the presented study, VO_2max_ increased without changes in blood oxygen capacity being observed. These results are consistent with earlier reports from studies where the IHT protocol was used (Katayama et al., 2004; Rodriguez et al., 2004; Roels et al., 2005; Dufour et al., 2006; Zoll et al., 2006; Czuba et al., 2011). Changes in blood oxygen capacity were not noted because the exposure to hypoxia was too short and therefore, insufficient to induce erythropoiesis (Czuba et al., 2011). Knaupp et al. (1992) who investigated the relationship between the duration of exposure to hypoxia and serum EPO concentration found that a 60-minute exposure to hypoxia did not significantly raise EPO concentration; its considerable increase (+50%) was only recorded after 120 minutes of hypoxia (F_I_O_2_= 10.5%). In a more recent study, Rodriguez et al. (2000) found that a regular 90-minute exposure to an altitude of 4000–5000 m (504 – 540 hPa) suffices to induce the secretion of EPO. The available reports (Gough et al., 2012) on the LH-TL method indicate, however, that only an exposure of 11–12 hours a day can effectively stimulate erythropoiesis.

The above studies lead together to a conclusion that LH-TL and IHT combined into a single training protocol can improve exercise capacity in normoxia. This concept was tested in the most recent study conducted by Robertson et al. (2010), who used LH-TL and IHT at the same time. The study involved highly-trained runners who were found to have significantly better VO_2max_ and exercise capacity at sea level measured by the 3 km running time (by respectively 4.8% and −1.1%) right after hypoxic training was completed. It is worth noting that although IHT alone also significantly increased VO_2max_ (by 2.2%), better running time was not recorded. Therefore, an appropriate combination of LH-TL and IHT can contribute to significant improvements in aerobic capacity and exercise performance in normoxia, particularly that the improvements come from distinctive adaptive mechanisms.

In the present research, a significantly smaller increase in blood lactate concentration (ΔLA) and a markedly smaller decline in blood pH (ΔpH) during the maximal exercise test after IHT were also observed, although the duration of exercise was considerably longer. However, the decrease in lactate concentration after the 12 minutes of recovery (ΔLA12′rec) was significantly higher after the IHT protocol compared to training in normoxia. This phenomenon is probably caused by changes in the concentration of membrane transport proteins, which follow from the exposure to hypoxia or altitude. Clark et al. (2004) measured in their experiment the concentration of MCT1 and MCT4 proteins in the skeletal muscles of highly-trained athletes that followed the LH-TL training for 20 nights. The experiment showed the rate of blood lactate release to be slower, but the amounts of MCT1 and MCT4 were not found to change. This may be attributed to the fact that the lactate /H^+^ transport system is activated more intensively during exercise than at rest (Juel et al., 2003). Zoll et al. (2006) demonstrated a 44% increase in the level of MCT1 mRNA in the skeletal muscles of nine well-trained runners after the IHT protocol was applied (3000 m ASL, twice a week, 24–40 minutes a day). In the control group that exercised in normoxia similar changes were not found. In the IHT group, the running time in the maximal exercise test was longer, without any changes in the maximum blood lactate concentration. Zoll et al. (2006) also noticed that the increased MCT1 mRNA improved lactate exchange and utilisation, which may have decelerated the pH decline during the running test, enabling the subjects to exercise longer at the set speed (Zoll et al., 2006).

The ability to regulate pH is known to be dependent not only on the concentration and activation of MCT1 and MCT4 proteins, but also on the activity of carbonic anhydrase (CA) (Juel et al., 2003). CA functions as a recipient or a donor of H^+^, and the reaction mediated by this enzyme may be amplified up to 10 million times (10^7^) to regulate how fast H^+^ and HCO_3_^−^ are transported. The research of Juel et al. (2003) indicates that 8 weeks of adaptation to high-altitude conditions (hypoxia) increased the amount of CA (IV) in glycolytic fibres. Another finding pointed to a greater amount of CA (IV) mRNA in the skeletal muscles after 6 weeks of hypoxic training (IHT) (Zoll et al., 2006). The above studies show therefore that adaptation to hypoxia can change the amount of transport proteins involved in the buffering process.

The results obtained by the authors during earlier studies (Czuba et al., 2011) and this research point to significantly lower maximum heart rate (HR_max_). The results obtained by Liu et al. (1998) are consistent with this assumption, as they point to significantly greater cardiac output (determined from echocardiography) in subjects that followed the LH-TL protocol. Liu et al. (1998) suggest that the cardiac output improves because of better contractility of the left ventricle. Other researchers noted similar changes in the subjects (Svedenhag et al., 1997; Miyazaki and Sakai, 2000). Why the muscle mass of the left ventricle should increase is not clear, but it is hypothesised that hypoxia stimulates the myocardium through stronger sympathetic activation of the heart, thereby leading to increased cardiac output (Wolfel et al., 1994).

## Conclusions

The most important finding of this work states that a 3-weekly intermittent hypoxic training protocol with high intensity intervals (4 or 5×4-min bouts at 90% of vVO_2max_-hyp) is an effective training means for improving aerobic capacity at sea level. The discrepancies between the outcomes of studies into IHT impacts on aerobic capacity and exercise capacity in normoxia are probably determined by the use of dissimilar and relatively incomparable methodological procedures, by the subjects having different training experience, and by different responsiveness of their bodies to hypoxic stimuli. The presented research is also a valuable enhancement to earlier reports on the effects of intermittent hypoxic training.

## Figures and Tables

**Table 1:** Average values of body mass and chosen variables of body composition in hypoxic (H) and control (C) groups during the experiment; *** - p<0.001, **-p<0.01, *- p<0.05

Variable	**Hypoxic (H) group**		**Control (C) group**	

	Before training	After training	Before training	After training

	n=6 x ± SD	n=6 x ± SD	n=6 x ± SD	n=6 x ± SD
Body height (cm)	188.8±6.1	-	194.3±6.6	-
Body mass (kg)	83.9±7.2	82.9±7.3	99.9±11.1	98.7±11.2
BMI (kg/m^2^)	23.5±1.5	23.3±1.6	26.3±1.6	26.0±1.5
Fat mass (kg)	9.3±2.5	9.1±3.5	11.6±4.6	11.1±3.4
Fat %	11.2±3.1	11.1±4.1	11.0±4.9	10.4±2.8

**Table 2:** Average values of the analyzed hematological variables in hypoxic (H) and control (C) groups during the experiment; *** - p<0.001, **-p<0.01, *- p<0.05

Variable	**Hypoxic (H) group**		**Control (C) group**	

	Before training	After training	Before training	After training

	n=6 x ± SD	n=6 x ± SD	n=6 x ± SD	n=6 x ± SD
WBC (10^3^/μl)	5.91±1.6	6.28±1.9	5.46±1.5	5.58±1.5
RBC (10^6^/μl)	5.08±0.34	5.11±0.44	4.82±0.2	4.92±0.1
HGB (g/dl)	15.4±0.6	15.6±0.7	15.1±0.6	15.3±0.6
HCT (%)	45.4±1.4	45.4±2.0	43.9±1.9	44.0±1.5

**Table 3:** Average values of considered variables registered during the ramp test in the hypoxic (H) and control (C) groups, as well as the significance of differences between both series of testing during the experiment ; *** - p<0.001, **-p<0.01, *- p<0.05

Variable	**Hypoxic (H) group**		**Control (C) group**	

	Before training	After training	Before training	After training

	n=6 x ± SD	n=6 x ± SD	n=6 x ± SD	n=6 x ± SD
Total distance (m)	**2644.6±489.8**	2909.3±470^***^	2313.2±379.8	2407.3±379.7
WR_max_ (W)	**380.3±55.1**	397.6±50.5^**^	426.1±20.8	433.5±21
WR_max_ (W/kg)	**4.52±0.36**	4.8±0.34^**^	**4.30±0.43**	4.42±0.47 *
VO_2max_ (ml/min)	**4416.3±461.6**	4701.6±467.2^***^	**5247.3±397.6**	5314.3±384.2 *
VO_2max_(ml/kg/min)	**52.6±3.9**	56.7±4.1^***^	**53±5.2**	54.1±5.2 *
RER_max_	1.1±0.03	1.09±0.02	1.07±0.01	1.08±0.02
VE_max_ (l/min)	154.9±16.9	155.3±11.6	170.4±17.9	171.2±18.5
BF_max_ (1/min)	59.6±7.1	59.7±8.4	55.4±7.9	55.2±7.7
HR_max_ (bpm)	**185±6**	182±5^*^	182±5	183±6
O_2_/HR_max_ (ml/bpm)	**23.8±2.9**	25.8±2.9^***^	28.9±2.1	29±2.0
ΔLA (mmol/l)	**8.25±1.63**	7.46±1.13^**^	8.58±1.60	8.37±1.73
ΔLA 12′rec (mmol/l)	**−2.12±0.88**	−2.57±0.77^*^	−2.08±0.53	−2.17±0.57
ΔpH	−0.181±0.041	−0.165±0.037	−0.174±0.028	−0.162±0.029

## References

[b1-jhk-39-103] Abdelkrim BN, El Fazaa S, El Ati J (2007). Time-motion analysis and physiological data of elite under-19-year-old basketball players during competition. Br. J. Sports Med.

[b2-jhk-39-103] Bunn HF, Poyton RO (1996). Oxygen sensing and molecular adaptation to hypoxia. Physiol Rev.

[b3-jhk-39-103] Bonetti DL, Hopkins WG (2009). Sea-level exercise performance following adaptation to hypoxia: a meta-analysis. Sports Med.

[b4-jhk-39-103] Castagna C, Abt G, Manzi V, Annino G, Padua E, D’Ottavio S (2008). Effect of recovery mode on repeated sprint ability in young basketball players. J. Strength Cond. Res.

[b5-jhk-39-103] Clark SA, Aughey RJ, Gore CJ, Hahn AG, Townsend NE, Kinsman TA (2004). Effects of live high, train low hypoxic exposure on lactate metabolism in trained humans. J. Appl. Physiol.

[b6-jhk-39-103] Czuba M, Waskiewicz Z, Zajac A, Poprzecki S, Cholewa J, Roczniok R (2011). The effects of intermittent hypoxic training on aerobic capacity and endurance performance in cyclists. J. Sports Sci. Med.

[b7-jhk-39-103] Desplanches D, Hoppeler H (1993). Effects of training in normoxia and normobaric hypoxia on human muscle ultrastructure. Pflügers Arch. Eur. J. Physiol.

[b8-jhk-39-103] Dufour SP, Ponsot E, Zoll J, Doutreleau S, Lonsdorfer-Wolf E, Geny B, Lampert E, Flück M, Hoppeler H, Billat V, Mettauer B, Richard R, Lonsdorfer J (2006). Exercise training in normobaric hypoxia in endurance runners. I. Improvements in aerobic performance capacity. J. Appl. Physiol.

[b9-jhk-39-103] Gough CE, Saunders PU, Fowlie J, Savage B, Pyne DB, Anson JM, Wachsmuth N, Prommer N, Gore CJ (2012). Influence of altitude training modality on performance and total haemoglobin mass in elite swimmers. Eur. J. Appl. Physiol.

[b10-jhk-39-103] Green H, MacDougall J, Tarnopolsky M, Melissa NL (1999). Downregulation of Na+-K+-ATPase pumps in skeletal muscle with training in normobaric hypoxia. J. Appl. Physiol.

[b11-jhk-39-103] Hamlin MJ, Marshall CH, Hellemans J, Ainslie PN, Anglem N (2010). Effect of intermittent hypoxic training on a 20 km time trial and 30 s anaerobic performance. Scan. J. Med. Sci. Sports.

[b12-jhk-39-103] Hendriksen IJM, Meeuwsen T (2003). The effect of intermittent training in hypobaric hypoxia on sea-level exercise: a cross-over study in humans. Eur. J. Appl. Physiol.

[b13-jhk-39-103] Helgerud J, Høydahl K, Wang E, Karlsen T, Berg P, Bjerkå M, Simonsen T, Helgesen C, Hjort N, Back R, Hoff J (2007). Aerobic high-intensity intervals improve VO2max more than moderate training. Med. Sci. Sports Exerc.

[b14-jhk-39-103] Juel C, Lundby C, Sander M, Calbet JA, Hall G (2003). Human skeletal muscle and erythrocyte proteins involved in acid–base homeostasis: adaptations to chronic hypoxia. J. Physiol.

[b15-jhk-39-103] Katayama K, Sato K, Matsuo H, Ishida K, Iwasaki K, Miyamura M (2004). Effect of intermittent hypoxia on oxygen uptake during submaximal exercise in endurance athletes. Eur. J. Appl. Physiol.

[b16-jhk-39-103] Knaupp W, Khilnani S, Sherwood J, Scharf S, Steinberg H (1992). Erythropoietin response to acute normobaric hypoxia in humans. J. Appl. Physiol.

[b17-jhk-39-103] Liu Y, Steinacker JM, Dehnert C, Menold E, Baur S, Lormes W (1998). Effect of living high-training low on the cardiac functions at sea level. Inter J Sports Med.

[b18-jhk-39-103] Melissa L, Macdougall JD, Tarnopolsky MA, Cipriano N, Green HJ (1997). Skeletal muscle adaptations to training under normobaric hypoxic versus normoxic conditions. Med Sci Sports Exerc.

[b19-jhk-39-103] McInnes SE, Carlson JS, Jones CJ, McKenna MJ (1995). The physiological load imposed on basketball players during competition. J. Sports Sci.

[b20-jhk-39-103] Miyazaki S, Sakai A (2000). The effect of “living high-training low” on physical performance in rats. Int. J. Biometeorol.

[b21-jhk-39-103] Morton JP, Cable NT (2005). Effects of intermittent hypoxic training on aerobic and anaerobic performance. Ergonomics.

[b22-jhk-39-103] Robertson EY, Saunders PU, Pyne DB, Gore CJ, Anson JM (2010). Effectiveness of intermittent training in hypoxia combined with live high/train low. Eur. J. Appl. Physiol.

[b23-jhk-39-103] Rodriguez FA, Truijens MJ, Townsend NE, Martini ER, Stray-Gundersen J, Gore CJ, Levine BD (2004). Effects of four weeks of intermittent hypobaric hypoxia on sea level running and swimming performance. Med. Sci. Sports Exerc.

[b24-jhk-39-103] Rodriguez FA, Ventura JL, Casas M, Casas H, Pages T, Rama R, Ricart A, Palacios L, Viscor G (2000). Erythropoietin acute reaction and haematological adaptations to short, intermittent hypobaric hypoxia. Eur. J. Appl. Physiol.

[b25-jhk-39-103] Roels B, Bentley DJ, Coste O, Mercier J, Millet GP (2007). Effects of intermittent hypoxic training on cycling performance in well-trained athletes. Eur J Appl Physiol.

[b26-jhk-39-103] Roels B, Millet GP, Marcoux CJL, Coste O, Bentley DJ, Candau RB (2005). Effects of hypoxic interval training on cycling performance. Med. Sci. Sports Exerc.

[b27-jhk-39-103] Svedenhag J, Piehl-Aulin K, Skog C, Saltin B (1997). Increased left ventricular muscle mass after long-term altitude training in athletes. Acta Physiol. Scand.

[b28-jhk-39-103] Terrados N, Jansson E, Sylvén C, Kaijser L (1990). Is hypoxia a stimulus for synthesis of oxidative enzymes and myoglobin?. J. Appl. Physiol.

[b29-jhk-39-103] Truijens MJ, Toussaint HM, Dow J, Levine BD (2003). Effect of high-intensity hypoxic training on sea-level swimming performances. J. Appl. Physiol.

[b30-jhk-39-103] Vallier JM, Chateau P, Guezennec CY (1996). Effects of physical training in a hypobaric chamber on the physical performance of competitive triathletes. Eur. J. Appl. Physiol. Occup. Physiol.

[b31-jhk-39-103] Ventura N, Hoppeler H, Seiler R, Binggeli A, Mullis P, Vogt M (2003). The response of trained athletes to six weeks of endurance training in hypoxia or normoxia. Int. J. Sports Med.

[b32-jhk-39-103] Vogt M, Puntschart A, Geiser J, Zuleger C, Billeter R, Hoppeler H (2001). Molecular adaptations in human skeletal muscle to endurance training under simulated hypoxic conditions. J. Appl. Physiol.

[b33-jhk-39-103] Wolfel EE, Selland MA, Mazzeo RS, Reeves JT (1994). Systemic hypertension at 4,300 m is related to sympathoadrenal activity. J. Appl. Physiol.

[b34-jhk-39-103] Zoll J, Ponsot E, Dufour S, Doutreleau S, Ventura-Clapier R, Vogt M, Hoppeler H, Richard R, Fluck M (2006). Exercise training in normobaric hypoxia in endurance runners. III. Muscular adjustments of selected gene transcripts. J. Appl. Physiol.

